# A new Approach to El Niño Prediction beyond the Spring Season

**DOI:** 10.1038/srep16782

**Published:** 2015-11-25

**Authors:** Shuhei Masuda, John Philip Matthews, Yoichi Ishikawa, Takashi Mochizuki, Yuusuke Tanaka, Toshiyuki Awaji

**Affiliations:** 1 Research and Development Center for Global Change, Japan Agency for Marine-Earth Science and Technology (JAMSTEC), Yokosuka 237-0061, Japan; 2 Environmental Satellite Applications, Llys Awel, Mount Street, Menai Bridge LL595BW, UK; 3 Institute of Liberal Arts and Sciences, Kyoto University, Kyoto 606-8501, Japan; 4 The Center for Earth Information Science and Technology, JAMSTEC, Yokohama 236-0001, Japan; 5 Project Team for Risk Information on Climate Change, JAMSTEC, Yokohama 236-0001, Japan; 6 Headquarters, Kyoto University, Kyoto 606-8501, Japan

## Abstract

The enormous societal importance of accurate El Niño forecasts has long been recognized. Nonetheless, our predictive capabilities were once more shown to be inadequate in 2014 when an El Nino event was widely predicted by international climate centers but failed to materialize. This result highlighted the problem of the opaque spring persistence barrier, which severely restricts longer-term, accurate forecasting beyond boreal spring. Here we show that the role played by tropical seasonality in the evolution of the El Niño is changing on pentadal (five-year) to decadal timescales and thus that El Niño predictions beyond boreal spring will inevitably be uncertain if this change is neglected. To address this problem, our new coupled climate simulation incorporates these long-term influences directly and generates accurate hindcasts for the 7 major historical El Niños. The error value between predicted and observed sea surface temperature (SST) in a specific tropical region (5°N–5°S and 170°–120°W) can consequently be reduced by 0.6 Kelvin for one-year predictions. This correction is substantial since an “El Niño” is confirmed when the SST anomaly becomes greater than +0.5 Kelvin. Our 2014 forecast is in line with the observed development of the tropical climate.

El Niño represents the dominant year-to-year climate variability in the tropical Pacific and exerts a wide range of influences over much of the globe^1^. It can trigger abnormal weather conditions, such as drought, in regions far from the tropics^2^,^3^ and it impacts greatly on marine ecosystems, including those associated with productive fisheries^1^. The largest recorded El Niño event of 1997/98 was indirectly responsible for the deaths of over 20,000 people and caused 34–45 billion US dollars’ worth of damage^4^. Climate-forecasting centers worldwide have therefore been making great efforts to enhance the accuracy of El Niño–Southern Oscillation (ENSO) predictions (see, for example, http://www.ecmwf.int/products/forecasts/d/charts, http://www.elnino.noaa.gov/forecast.html, http://ds.data.jma.go.jp/gmd/tcc/tcc/products/elnino/index.html)^5^,^6^. Although this work has largely been based on state-of-the-art atmosphere-ocean models and data assimilation approaches^7^, prediction success has been mixed. Most recently, climate forecasters heralded 2014 as a special year, since they anticipated the development of a major El Niño^8^,^9^. Several signs of onset were detected in both ocean surface and subsurface parameters^10^, though by northern winter the 2014 El Niño had still not arrived. Although there are a range of views as to the cause of this mis-prediction, the climate-research community has yet to reach a consensus on what went wrong. Uncertainty will thus still remain in future predictions.

Much of the difficulty stems from an intractable problem termed the “spring persistence barrier” (SPB), which tends to cause forecast skill to drop abruptly when projections are made through boreal spring^11^,^12^. The seasonal dependence of forecast skill has therefore been the subject of many studies^13^,^14^,^15^ and thorough review^16^.

Some of this work investigates the possible mechanisms causing abrupt reductions in skill by using a coupled model and reveals the influence of initial Sea Surface Temperature (SST) errors on the predictions^17^,^18^, and the possible role of assimilation of wind observations in enhancement of the forecast skill through more accurate initialization^19^. The lack of semi-stochastic, relatively short-term wind variations in coupled model systems is another cause of degradation in relatively short-term ENSO forecasts^20^. Recent work shows that the seasonal dependence of ENSO growth rate can cause a seasonal dependence in forecast skill^21^. Nevertheless, the factors causing loss in predictive capability have not been thoroughly uncovered, partly because these model results are difficult to validate with limited observations. Given this background, we have focused on a data assimilation approach to analyze the temporal changes of climate state and reduce the abrupt drop in forecast skill that develops as a result of the SPB^22^.

## Results

### Modulated Seasonal Variability in Tropical Climate System

As its name suggests, the SPB stems from interactions between the El Niño and seasonal cycles of tropical climate system. In addition, previous literature showing that the dominance of the SPB evolves on a decadal timescale is quite suggestive^23^. Typically, forecast skill drops in spring for predictions made in the 1970s, while such seasonal dependence is rather small for 1980 s predictions. This decadal change is related to long-term variations found in the timing of sea surface temperature evolution at the development phase of El Niño events and implies that the state of the ocean-atmosphere coupling is central to revealing the cause of the diminution in forecast skill associated with the SPB. Here, we assess the temporal evolution of an important variable in the energetics of the El Niño and La Niña on the basis of a reconstructed tropical climate state covering the past 47 years (Methods). This climate state is reconstructed from a coupled data assimilation system based on a 4-dimensional variational approach, in which the adjoint form of the atmospheric and oceanic general circulation models are applied to seek the best temporal trajectory of the model variables consistent with the observational data^22^. The obtained temporal evolution of the ocean-atmosphere climate state is realistic and dynamically-self consistent, and thus ideally suited to analyze El Niño dynamics.

**Figure 1:**
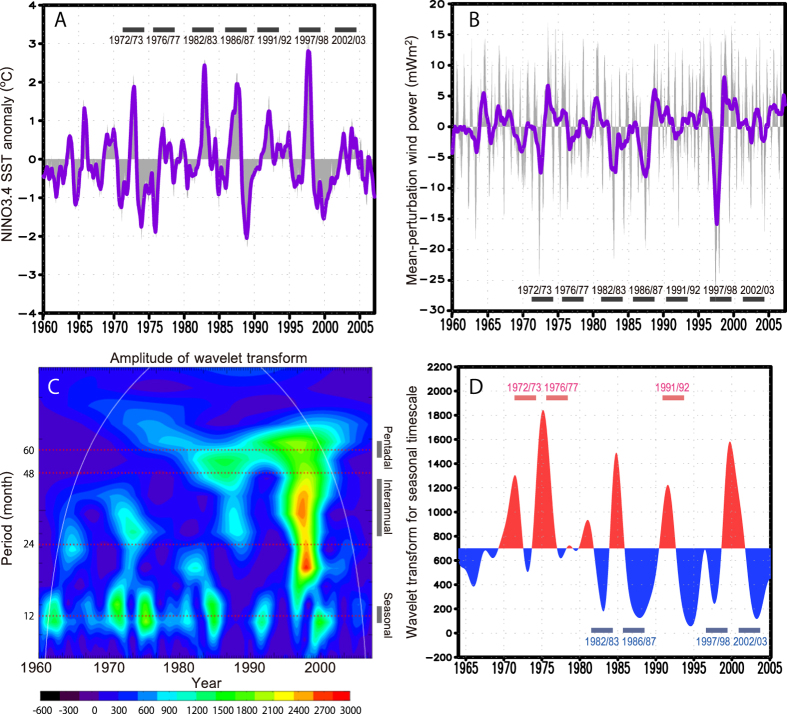
Reconstructed time series of the tropical climate state for the period 1960–2006. (**A**) Sea surface temperature anomaly averaged in the NINO3.4 region (170^o^-120^o^W, 5^o^S-5^o^N). Gray shade denotes 10-daily mean time series. Violet curve shows 3-month running mean. Units are degrees Celsius. Gray dashed line shows the periods of major El Niño events (see text). (**B**) Mean perturbation wind power (W_mp_)^24^ averaged in 150^o^E-100^o^W, 5^o^S-5^o^N. Units are mWm^−2^. Gray shade denotes 10-daily mean time series. Violet curve shows 1-year running mean. (**C**) Magnitude of wavelet transform of W_mp_. (**D**) Decomposed W_mp_ spectrum on seasonal timescale. Red and blue dashed lines show the periods of major El Niño events occurring in positive and negative decadal phase of seasonal variablity, respectively (see text).

Figure 1A exhibits the time series of the Sea Surface Temperature anomaly (SSTa) in the NINO3.4 region (5°N–5°S and 170°–120°W) of the reconstructed ocean-atmosphere coupled climate state for the period 1960–2006, which has often been used to generate an index for classifying ENSO conditions. A major El Niño is here defined as an event during which the five-month running mean of the NINO3 (5°N–5°S and 150°–90°W) SSTa exceeds +0.5 °C for 6 consecutive months (e.g., Japan Meteorological Agency; gray dashed lines). Figure 1B shows the time series of the mean perturbation wind power W_mp_ as defined by Goddard and Philander^24^ (W_mp_ ∝ *u′ <τ*> + *τ*′<*u*> where *τ* is the zonal wind stress, u surface zonal oceanic velocity, <x> denotes mean component of x, x′ its perturbation component; Supplementary Information (SI), S1), which represents the anomalous component of the work delivered by the atmosphere to the ocean in the tropical ocean (5°N–5°S and 150°E–100°W). An increase (decrease) of this parameter implies a steepening (flattening) in the zonal thermocline gradient, which in turn leads to greater likelihood of low (high) SSTa in the eastern tropical Pacific as La Niña (El Niño) events after approximately 3 months (Fig. 1A,B).

Figure 1C shows the amplitude of the wavelet transform for the time series of W_mp_ (Fig. 1B). Spectral peaks above the significance level (within white curve) are apparent at roughly seasonal (period of 12 month), interannual (period of 24–48 month), and longer timescales. The amplitudes of the variabilities within each waveband change dramatically from year to year. The power of pentadal and interannual variability is relatively large after the 1980 s when large ENSO events repeatedly occur. This is consistent with long-term modulation of ENSO phenomena^25^. The power of the seasonal variability is not constant and is likely modulated by variations on pentadal to decadal timescales (Fig. 1D), despite the regularity of insolation. The variation of the power shows positive anomaly phase in the 1970s, where the values are above a long-term mean value for the period of 1960–2006, and negative phase in the period 1980–2005, but with some short-term positive anomaly periods (e.g., the beginning of 1990 s). The relevant phases for the major El Niños are here assumed to be determined by the anomalous sign (red or blue color) of Fig. 1D at the start of the spring time through which each El Niño develops. These features are basically consistent with the decadal and seasonal dependence of ENSO prediction skill in 1970 s and 1980 s, as reported in the previous literature^23^, where the conventional analysis of NINO3.4 SST shows that its variance of seasonality changes on a decadal timescale. Recent work^26^ shows that such decadal modulation in seasonality is also found in the relationship between thermocline depth and SSTa in the eastern equatorial Pacific and that the linkage between surface and subsurface ocean variables around springtime becomes weak during the 1970 s and late 1990 s, which seemingly results in a robust SPB in these decades. This decadal dependency can be closely related to the variations in the annual exchange of kinetic energy between the atmosphere and ocean (Fig. 1D).

The interaction among seasonal, interannual, and longer-term variabilities is not obvious from this plot although some linkage seems to exist, for instance, the 1997–1998 El Niño is boosted by seasonal variability at the initial phase, possibly in conjunction with westerly wind-burst events.

Note that the seasonal variablity is statistically independent from interannual variations since the correlation coefficient between decomposed W_mp_ spectra on seasonal (Fig. 1D) and interannual timescales is −0.13, and thus has an absolute value that is lower than the critical r-value (0.27) with 41 degrees of freedom for 1965–2005.

The above results imply that the annual exchange of kinetic energy between the atmosphere and ocean responsible for ENSO genesis (Fig. 1B) is modulated on pentadal to decadal timescales largely independent of the inherent ENSO variability. This modulation could influence the seasonal dependence of ENSO prediction skill through mechanisms such as nonlinear interaction or dynamical combination^27^,^28^,^29^,^30^.

### Which Mechanisms are Responsible for the Modulation?

**Figure 2:**
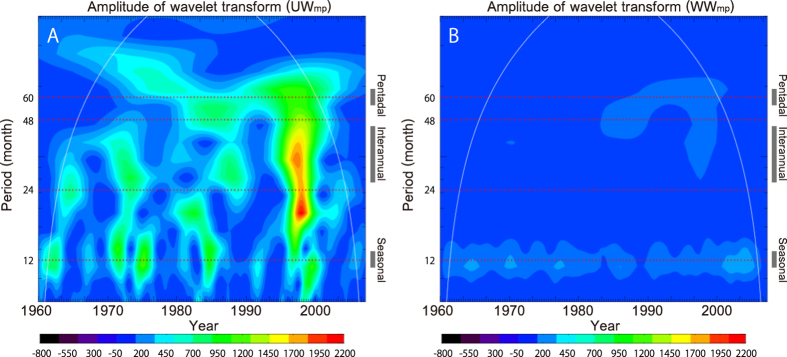
Magnitude of the wavelet transform of each component of the mean perturbation wind power. (**A**) Adjustment wind power UW_mp_ (*u*′*<τ*>). (**B**) Mature event wind power WW_mp_ *τ*′*<u>*^24^.

The mean perturbation wind power, W_mp_ is composed of two elements with different physical origins. The first term is related to surface zonal velocity change in conjunction with oceanic adjustments made mainly by radiating long oceanic waves (UW_mp_ = *u′<τ*>)^24^, implying that it is related to the cyclic evolution of ENSO, similar in nature to the operation of a delayed oscillator^31^. The other term is related to the anomalous wind forcing (WW_mp_ = τ′<u>) and is associated ENSO genesis resulting from atmospheric perturbations^32^. Figure 2 shows the results of wavelet analysis for each component of W_mp_. Consistent with the previous literature^24^, UW_mp_ dominates the W_mp_ for almost all wavelengths (Figs 2A and 1C). This implies that oceanic adjustment plays an essential role in ENSO evolution. In this regime, the relatively long oceanic memory for climate change leads to high predictability. On the other hand, although WW_mp_ is negligible when compared with UW_mp_, it is apparently visible at times of seasonal variability (Fig. 2B). The long-term modulation of the seasonality in W_mp_ thus stems from the interplay of variations in UW_mp_ and WW_mp_ and, in particular, the pentadal/decadal change in the WW_mp_ term. Hence, long-term modulation of the annual anomalous wind stress in the tropical regions plays an important role in determining the trend in W_mp_. This implies that the representation of wind stress variability during ENSO genesis is vital factor for an accurate ENSO prediction^19^.

One possible causative mechanism of long-term modulation in the wind field can be found in the non-linear dynamics of tropical climate system^27^,^28^. Conceptual model experiments show the possibility that atmospheric nonlinear interactions can take place between the mechanisms causing seasonal and inter-annual variation and lead to long-term modulation of the tropical seasonality (SI, S2; Fig. S1). A further possible cause is the influence of long-term climate change in the tropical ocean, for instance, known as Tropical Pacific Decadal Variability^33^,^34^. Although the phase of this decadal variability, which changed once around 1980 during 1965–1997^33^, does not exactly match the phase of long-term modulation of the seasonality of WW_mp_ (or W_mp_), the fact that negative phases of modulation appear more frequently after 1982 is suggestive of a link.

These possible mechanisms can interact to produce subtle modulations. Nevertheless, the history of systematic ocean observations is still too short to determine which of these mechanisms is responsible for the long-term modulation. Sustained monitoring in the tropical ocean will be required to resolve this issue.

#### New El Niño Forecasting through Control of the Atmosphere-Ocean Coupling Parameters

None of the prediction models currently in use explicitly incorporate long-term changes in the annual surface energy exchange associated with El Niño evolution. They are, however, included implicitly, in part because their influences on the genesis of El Niño are poorly understood. We consider this omission to represent a fundamental weakness in efforts to make more accurate prediction*s* beyond boreal spring.

**Figure 3:**
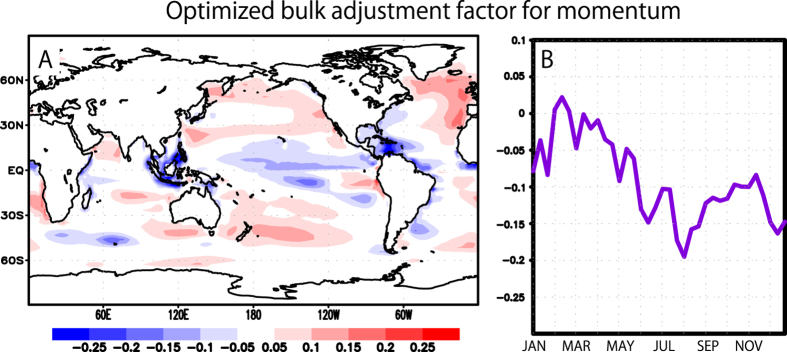
The optimized bulk adjustment factor for wind stress. (**A**) The spatial distribution of the annual average in logarithmic scale^22^. (**B**) The temporal change averaged in NINO3.4 region. The map is generated by Grid Analysis and Display System (GrADS) version 2.0 (http://iges.org/grads/).

Our state-of-the-art ocean-atmosphere coupled data assimilation system generates time series of the optimal “adjustment factors” required for empirical correction of the coupling intensity assumed in the model^22^. The adjustment factors are essential for the correct regulation of heat, fresh water, and momentum exchange through the sea surface. We make use of these values in order to incorporate the modulation effects shown in Fig. 3, where the spatial distribution is largely similar to that presented in the thorough investigation of Sugiura *et al.*^22^ (Fig. 3A). The temporal change shows that the adjustment factor works to relatively enhance energy exchange from January to May for the equatorial region (Fig. 3B), which is again consistent with the results of Sugiura *et al.*

We start by constructing a set of seasonal adjustment factors from the climatology by simply averaging the historical values of the optimal adjustment factor which are calculated over the 27-year period from 1980 to 2006. There is no loss of generality in choosing the averaging decades. Then, we identify which phase of the pentadal to decadal cycle in the tropical seasonal state is appropriate on the basis of the estimated time series of W_mp_ (Fig. 1D). Under the assumption that long-term modulations continue along their recent trend within a few years of prediction, we determine the values of the appropriate adjustment factor for the future projection. For practical use, we simply apply the adjustment factor as either a “0” or a “1”, depending on the negative or positive sign of the anomaly of the W_mp_ seasonality relative to the long-term mean value. Factors of 0 are relevant to periods with relatively weak seasonal variations in energy exchange, such as frequently appeared from the 1980 s onward (negative anomaly periods; blue shade in Fig. 1D), and in which the modeled coupling intensity agrees with the conventional bulk formulae without seasonal control. This means that no adjustment to the modeled coupling intensity is required for these periods. On the other hand, a factor of 1 should be applied to period*s* with strong seasonality such as in the 1970 s (positive anomaly periods; red shade in Fig. 1D), so that the modeled coupling intensities are boosted by their respective seasonal adjustment factor. Thus, we explicitly adjust forecasts covering periods of high seasonal variability, whereas forecasts for periods of weak seasonal variability are left unadjusted.

### Predictability of Recent Historical El Niño’s

**Figure 4:**
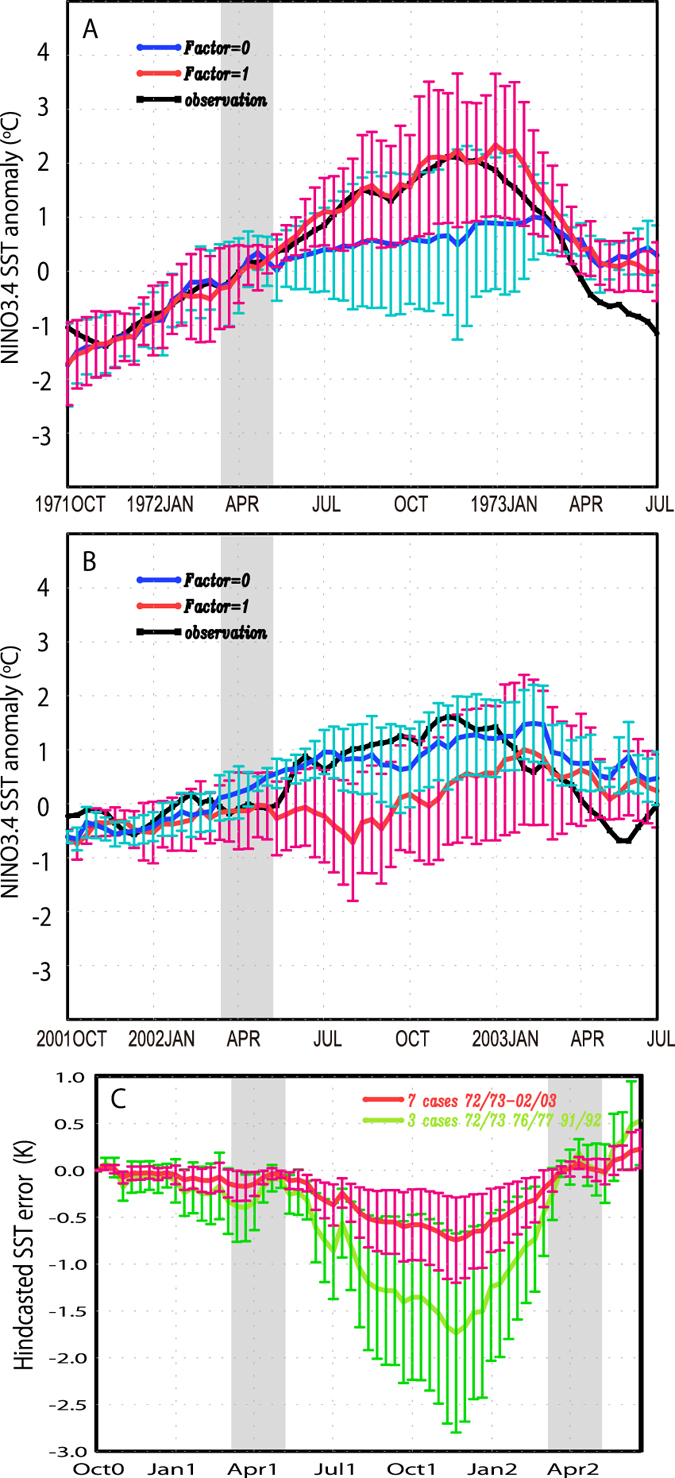
Predicted NINO 3.4 SST anomaly (red and blue curve) as compared with observed SST anomaly (black)^46^ and the accuracy of prediction. (**A**) The 10-daily values are for hindcast experiments during 1971–1973 derived from the case without seasonal control (Factor = 0: blue) and the optimized case using a seasonal adjustment factor (Factor = 1: red). Bars show errors estimated from standard deviation values of the ensemble forecasts. Grey shaded regions denote the boreal spring periods. (**B**) The same as (**A**) but for hindcast experiments during 2001–2003. Units are degrees Celsius. (**C**) Prediction error estimated by difference in root mean square differences for hindcasted NINO3.4 SSTs between conventional and advanced predictions for 7 El Nino events (red) and 3 events in a period of strong seasonality (green). Units are in kelvin.

To examine the effectiveness of our new scheme, we have executed 2.5-year ensemble hindcast experiments (Methods) for all the past major El Niño episodes after 1970 (Fig. 1A). Figure 4 shows the time series of predicted SST averaged over the NINO3.4 area for the periods of major El Niño events, firstly for 1972–1973, which covers a period of relatively strong seasonal variations (Fig. 1D), and then for 2002–2003 (Fig. 4B), covering a period with relatively weak seasonal variations (Fig. 1D). The forecast for the 1972–1973 El Niño using an adjustment factor of value 1 produces more accurate results (red curve in Fig. 4A) than the forecast with this factor set to 0 (blue curve). In particular, the latter shows an unrealistic temperature drop immediately after spring 1972 (after gray shaded region in Fig. 4A). This result demonstrates that control of the annual energy exchange in accordance with long-term modulation in the real climate system is a vital factor, albeit one missing from conventional ENSO prediction schemes. In short, the decay in forecast skill associated with the SPB cannot be resolved if such long-term influences are neglected.

In contrast, the 2002–2003 El Niño event occurred during a period with relatively weak seasonal variations. Our results show better forecast skill for this event when used with an adjustment factor of 0 (blue curve in Fig. 4B). The time series derived with the factor set to 1 exhibits a different temporal evolution in SST after the SPB period (red curve). These results are again consistent with the adopted scenario of a long-term modulation in seasonal variability. Further, confirmation was derived from other hindcast experiments for all the major El Niño events from 1975 to 1998 (SI, Fig. S2), although the 1991–1992 El Niño is a marginal case for which the phase likely changes during 2.5-year hindcast (Fig. S2D).

We have also calculated the root mean square difference (rmsd) in NINO3.4 SST between forecast results and observations for all hindcast experiments (7 cases after 1970) in order to reveal the broader advantages of controlling the annual coupling intensity to reflect the phase of the long-term modulation (Fig. 1D). Figure 4C shows differences between the rmsd for cases when the adjustment factors are well chosen and for the conventional cases without seasonal control. The minus values indicate error reduction and hence larger minus values mean more effective error reduction and eventually better El Niño predictions. Minus values continue after the SPB period. The value after 1 year (Oct1) from the start of the forecast (Oct0) is −0.6 kelvin (red curve). When we focus solely on 3 cases, each of relatively strong seasonal variation (i.e., 1972–1973, 1976–1977, and 1991–1992), the error reduction for the one-year prediction reaches 1.3 kelvin (green curve). These improvements in El Niño prediction capability after the SPB period are therefore significant since the “El Niño” is often confirmed when the SST anomaly becomes greater than +0.5 Kelvin.

It is well known that the initialization procedure can sometimes have a determining influence on El Niño predictions over timescales of several months^13^,^35^. However, the establishment of optimized initial conditions alone cannot resolve the decay in forecast skill associated with the SPB in cases when the energy exchange at the development phase is not properly resolved, as is particularly the case in and after boreal spring. Our El Niño prediction scheme should then be used since it greatly advances the control of the modeled seasonal variability when external information on the long-term modulation is properly determined. In this study, we have considered 7 major El Niño events. These in fact represent the sum total of cases presently available for analysis as coupled data assimilation products. More events and case studies are of course required - not only in terms of data acquisition for future/past El Niños but also through analyses based on other model architectures. However, our approach to stemming the diminution in forecast skill in boreal spring is new in that it incorporates a time-dependent adjustment factor whose influence can lead to more realistic forecasts as a result of better representation of dynamical interactions taking place on multiple timescales.

### Near-term Fate of the Tropical Climate System

**Figure 5:**
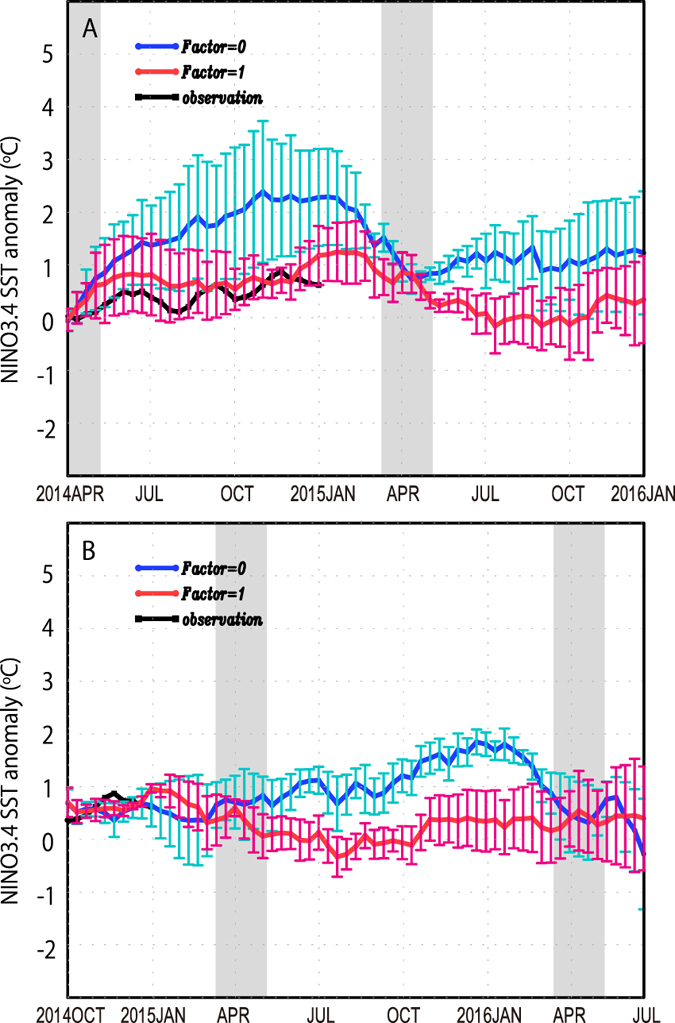
Predicted NINO 3.4 SST anomaly (red and blue curve) as compared with observed SST (black) for the period of 2014–2016. The same as Fig. 4A,B but for forecast results starting from (**A**) April 2014 and (**B**) October 2014.

Finally we consider the forecast for 2014–2015 on the basis of our new scheme (SI, S3). The present phase of the decadal modulation, which is required in order to select the relevant adjustment factor, can be determined from the amplitude of the seasonal variability in the recent W_mp_. After 2007 the phase most likely entered a period corresponding to strong seasonal variations (SI, Fig. S3) and thus a factor of 1 can be assumed to be relevant for the 2014–2015 prediction. In contrast to most published reports based on a conventional mean (for instance, the blue curve in Fig. 5A), our system predicts no strong El Niño during 2014 (red curve), consistent with the tropical climate state in the winter season of 2014 (black line), and in stark contrast to the mis-predictions of other national climate centers^8^,^9^. Note that these projections start before the period of the SPB.

On longer timescales our system shows that the probability of a major El Niño event developing in the period up to winter 2015 is very low. Our projection initiated from July 1st 2014 (red curve in Fig. 5B) also shows that the probability that a neutral condition will last from April to winter 2015 is above 60%. If the current decadal phase abruptly switches at some point during 2015, our system indicates that the possibility of a major El Niño event during this year becomes high (blue curves in Fig. 5B).

It is noteworthy that the accuracy of our new prediction scheme depends on the accuracy with which we can detect the phase of pentadal/decadal modulation of seasonal variation in which the tropical climate system evolves. Recent climate research in the area of decadal phase detection/prediction has been successful, particularly for mid-latitude climate change^36^,^37^.

Although decadal phase prediction in the tropical regions remains uncertain, with a number of issues still to be overcome^38^, including the possible link to the other basins^39^,^40^,^41^, recent advances have been encouraging. For instance, improvement of the initialization schemes employed in atmosphere-ocean coupled systems can lead to more accurate phase transitions for the Pacific region^42^.

In line with these achievements, the diagnosis and short-term prediction of the intensity of the annual cycle, the key factor required for our El Niño prediction scheme, must be improved. Though work to date in this area is promising, further efforts are now necessary.

## Conclusions

We have argued that the influence of pentadal to decadal modulations on the annual surface-energy exchange responsible for El Niño genesis is a key factor in resolving the decay in forecast skill associated with the SPB, although it is not directly considered in any of the standard ENSO forecast models presently in use. By incorporating this important aspect our approach therefore provides a new, improved forecast capability in which the energetics of the climate cycle are represented in a realistic fashion. Clearly, in the light of these results, the unfolding of El Niño activity during the 2015 will provide a crucial test of current understanding of the phenomenon and will further challenge our predictive capabilities. A similar approach involving phase-related adjustment factors may in principle be applicable to La Niña predictions. Further work along these lines will lead to more reliable forecasts of ENSO including warm and cool events.

## Methods

### Data

A 47-year reconstructed climate state estimation was deduced from a coupled ocean-atmosphere data assimilation experiment. The data assimilation system was originally constructed within the JAMSTEC K7 consortium^22^ for use in climate research studies^43^,^44^.

The assimilation is based on a 4-dimensional variational adjoint approach, in which adjoint codes of the AGCM and OGCM are applied to seek the best possible temporal trajectory of the model variables on the basis of observational data. As a result, high-quality analysis fields are produced. The temporal evolution of the ocean-atmosphere coupled state is partly controlled by some of the coupling parameters in this system.

The parameters are determined through statistical optimization on the basis of model dynamics and observational data. These data are freely available in part, from the following web site, http://k7-dbase2.yes.jamstec.go.jp/las/servlets/dataset? catitem = 100.

In this paper, we use 10-daily data from this system. The mean value in our main analyses is determined by a 47-year 10-daily mean for each variable.

### Simulations

The 2.5-year ensemble hindcast experiments were executed on the Earth Simulator by using the K7 coupled ocean-atmosphere model^22^. The applied ocean general circulation model used a horizontal resolution of 1° in both latitude and longitude, with 45 vertical levels. The atmospheric general circulation models the commonly used T42 spectral model and has 24 layers in vertical σ coordinates.

The initial conditions for each forecast year were deduced from a coupled data assimilation experiment for the first nine months from January to September. The prediction therefore started in October of the first year. The initialization of oceanic variables is done for the 1^st^ January. The optimization of the coupling parameters are done for January-September every 10 days. Model runs are not explicitly bias-corrected as a result of fully coupled data assimilation approach^22^.

An ensemble experiment with five coupled model runs was performed, each of which used different atmospheric initial conditions determined by the Lagged Average Forecasting method^45^. The latter was applied separately using a time interval of 2 days, but with the same optimized oceanic initial conditions^22^.

## Additional Information

**How to cite this article**: Masuda, S. *et al.* A new Approach to El Niño Prediction beyond the Spring Season. *Sci. Rep.* **5**, 16782; doi: 10.1038/srep16782 (2015).

## Supplementary Material

Supplementary Information

## References

[b1] Philander S. G. Our Affair With El Niño: How We Transformed an Enchanting Peruvian Current into a Global Climate Hazard. Princeton Univ Press (2004).

[b2] Kovats R. S. El Niño and Human Health. Bull. W.H.O. 1127–1135 (2000). 11019461 PMC2560836

[b3] Kovats R. S. *et al.* El Niño and health. The Lancet 362, 1481–1489 (2003). 10.1016/S0140-6736(03)14695-8 14602445

[b4] Sponberg K. Compendium of Climatological Impacts. University Corporation for Atmospheric Research **1** (National Oceanic and Atmospheric Administration, Office of Global Programs) (1999).

[b5] Barnston A. G., Tippett M. K., L’Heureux M. L., Li S. & Dewitt D. G. Skill of Real-time Seasonal ENSO Model Predictions during 2002–2011: Is Our Capability Increasing? Bull. Amer. Meteor. Soc. 93, 631–651 (2012).

[b6] Climate Prediction Center/NCEP. ENSO: Recent Evolution, Current Status and Predictions. http://www.elnino.noaa.gov/ (2014). Date of access: 07/07/2014.

[b7] Masuda S. *et al.* Simulated Rapid Warming of Abyssal North Pacific Waters. Science 329, 319–322 (2010). 20576848 10.1126/science.1188703

[b8] Tollefson J. El Niño tests forecasters. Nature 508, 20–21 (2014). 24695295 10.1038/508020a

[b9] Bureau of Meteorology, Commonwealth of Australia. ENSO Wrap-Up. http://www.bom.gov.au/climate/enso/ (2014). Date of access: 08/04/2014.

[b10] Menkes C. E. *et al.* About the role of Westerly Wind Events in the possible development of an El Niño in 2014. Geophys. Res. Lett. 41, 6476–6483 (2014).

[b11] Wright P. B. Persistence of rainfall anomalies in the central Pacific. Nature 277, 371–374 (1979).

[b12] McPhaden M. J. Tropical Pacific Ocean heat content variations and ENSO persistence barriers. Geophys. Res. Lett. 30, 33-1–33-4 (2003).

[b13] Torrence C. & Webster P. J. The Annual Cycle of Persistence in the El Nino-Southern Oscillation. Q. J. Roy. Meteor. Soc. 124, 1985–2004 (1998).

[b14] Chen D. *et al.* Predictability of El Nino in the past 148 years. Nature 428, 733–736 (2004). 15085127 10.1038/nature02439

[b15] Jin E. K. *et al.* Current status of ENSO prediction skill in coupled ocean–atmosphere models. Climate Dynamics 31, 647–664 (2008).

[b16] Webster P. J. & Hoyos C. D. Beyond the spring barrier? Nature Geoscience 3, 152–153 (2010).

[b17] Mu M., Duan W. & Wang B. Season-dependent dynamics of nonlinear optimal error growth and El Nino-Southern Oscillation predictability in a theoretical model. J. Geophys. Res. 112, D10113, 10.1029/2005JD006981 (2007).

[b18] Duan W. & Wei C. The “spring predictability barrier” for ENSO predictions and its possible mechanism: Results from a fully coupled model. Int. J. Climatol. 33, 1280–1292 (2013).

[b19] Zheng F. & Zhu J. Coupled assimilation for an intermediated coupled ENSO prediction model. Ocean Dyn. 60, 1061–1073 (2010).

[b20] Lopez H. & Kirtman B. P. WWBS, ENSO predictability, the spring barrier and extreme events. J. Geophys. Res. Atmos. 119, 10114–10138, 10.1002/2014JD021908 (2014).

[b21] Levine A. F. Z. & McPhaden M. J. The annual cycle in ENSO growth rate as a cause of the spring predictability barrier. Geophys. Res. Lett. 42, 10.1002/2015GL064309 (2015).

[b22] Sugiura N. *et al.* Development of a 4-dimensional variational coupled data assimilation system for enhanced analysis and prediction of seasonal to interannual climate variations. J. Geophys. Res. 113, 10.1029/2008JC004741 (2008).

[b23] Balmaseda M. A., Davey M. K. & Anderson D. L. T. Seasonal dependence of ENSO prediction skill. J. Clim. 8, 2705–2715 (1995).

[b24] Goddard L. & Philander S. G. The energetics of El Nino and La Nina. J. Climate 13, 1496–1516 (2000).

[b25] Wittenberg A. T. Are historical records sufficient to constrain ENSO simulations? Geophys. Res. Lett. 36, L12702, 10.1029/2009GL038710 (2009).

[b26] Zhu J., Kumar A. & Huang B. The relationship between thermocline depth and SST anomalies in the eastern equatorial Pacific: Seasonality and decadal variations. Geophys. Res. Lett. 42, 4507–4515 (2015).

[b27] Timmermann A. & Jin F. F. A nonlinear mechanism for decadal El Niño amplitude changes. Geophys. Res. Lett. 29, 10.1029/2001GL013369 (2002).

[b28] Timmermann A. Decadal ENSO amplitude modulations: A nonlinear paradigm. Global Planet Change 37, 135–156 (2003).

[b29] Sun F. & Yu J. Y. A 10–15-yr modulation cycle of ENSO intensity. J. Clim. 22, 1718–1735 (2009).

[b30] Stuecker M. F. *et al.*, A combination mode of the annual cycle and the El Niño/Southern Oscillation. Nature Geoscience 6, 540–544 (2013).

[b31] Suarez M. J. & Schopf P. S. A delayed action oscillator for ENSO. J. Atmos. Sci. 45, 3283–3287 (1988).

[b32] Lengaigne M. *et al.* Triggering of El Niño by westerly wind events in a coupled general circulation model. Clim. Dyn. 23, 601–620 (2004).

[b33] Yeh S. W. & Kirtman B. P. Pacific decadal variability and decadal ENSO amplitude modulation. Geophys. Res. Lett. 32, L05703, 10.1029/2004GL021731 (2005).

[b34] Ogata T., Xie S. P., Wittenberg A. & Sun D. Z. Interdecadal Amplitude Modulation of El Niño–Southern Oscillation and Its Impact on Tropical Pacific Decadal Variability. J. Clim. 26, 7280–7297 (2013).

[b35] McPhaden M. J. *et al.* The Tropical Ocean-Global Atmosphere (TOGA) observing system: A decade of progress. J. Geophys. Res. 103, 14,169–14,240 (1998).

[b36] Sugiura N. *et al.* Potential for decadal predictability in the North Pacific region. Geophy. Res. Lett. 36, L20701, 10.1029/2009GL039787 (2009).

[b37] Mochizuki T. *et al.* Pacific decadal oscillation hindcasts relevant to near-term climate prediction. Proc. Natl. Acad. Sci. 107, 1833–1837 (2010). 20080684 10.1073/pnas.0906531107 PMC2804740

[b38] Wittenberg A. T. *et al.* ENSO modulation: Is it decadally predictable? J. Clim. 27, 2667–2681 (2014).

[b39] Luo J. J., Sasaki W. & Masumoto Y. Indian Ocean warming modulates Pacific climate change. Proc. Natl. Acad. Sci. 109, 18,701–18,706 (2012). 23112174 10.1073/pnas.1210239109 PMC3503235

[b40] McGregor S. *et al.* Recent Walker Circulation strengthening and Pacific cooling amplified by Atlantic warming. Nature Climate Change 4, 10.1038/nclimate2330 (2014).

[b41] Chikamoto Y. *et al.* Skilful multi-year predictions of tropical trans-basin climate variability. Nature Communications 6, 6869 10.1038/ncomms7869 (2015). PMC4410635 25897996

[b42] Ding H. *et al.* Hindcast of the 1976/77 and 1998/99 climate shifts in the Pacific. J. Clim. 26, 7650–7661 (2013).

[b43] Mochizuki T. *et al.* Improved coupled GCM climatologies for summer monsoon onset studies over southeast Asia. Geophys. Res. Lett. 34, 10.1029/2006GL027861 (2007).

[b44] Toyoda T. *et al.* A possible role for unstable coupled waves affected by resonance between Kelvin waves and seasonal warming in the development of the strong 1997-1998 El Nino. Deep-Sea Research 56, 495–512 (2009).

[b45] Hoffman R. N. & Kalnay E. Lagged average forecasting. Tellus Ser. A 35, 100–118 (1983).

[b46] Reynolds R. W. *et al.* An improved *in situ* and satellite SST analysis for climate. J. Climate 15, 1609–1625 (2002).

