# Supplementary material for: A new Approach to El Niño Prediction beyond the Spring Season
Source: Sci Rep. 2015 Nov 25;5:16782. doi: 10.1038/srep16782 (PMC4658476; doi:10.1038/srep16782)
Supplement: Supplementary Information [file srep16782-s1.pdf]

Supplementary Information for

A new Approach to El Niño Prediction

beyond the Spring Season

Shuhei Masuda[1,*]
John Philip Matthews[2,3]
Yoichi Ishikawa[4]
Takashi Mochizuki[5]
Yuusuke Tanaka[4]
Toshiyuki Awaji[6]

[1]Research and Development Center for Global Change, Japan Agency for Marine-Earth Science and Technology (JAMSTEC), Yokosuka 237-0061, Japan

[2]Environmental Satellite Applications, Llys Awel, Mount Street, Menai Bridge LL595BW, UK

[3]Institute of Liberal Arts and Sciences, Kyoto University, Kyoto 606-8501, Japan

[4]The Center for Earth Information Science and Technology, JAMSTEC, Yokohama 236-0001, Japan

[5]Project Team for Risk Information on Climate Change, JAMSTEC, Yokohama 236-0001, Japan

[6]Headquaters, Kyoto University, Kyoto 606-8501, Japan

*To whom correspondence should be addressed. E-mail: smasuda@jamstec.go.jp

S1: Mean perturbation wind power

Goddard and Philander (24) show that the mean perturbation power W_{mp} in the energetics of El Niño and La Niña is approximated as follows,

$$W_{\mathrm{mp}} \propto u'<\tau> + \tau'<u>$$

where τ is the zonal wind stress, u surface zonal oceanic velocity, <x> denotes mean component of x, x' its perturbation component. The values here were averaged over the tropical Pacific (5°S–5°N and 150°E–100°W). The quantity W_{mp} loosely represents the work done by the winds on the ocean except that for the maintenance of the climatological mean state.

S2: A possible long-term modulation in a phenomenological low-order conceptual model

The model was originally constructed as a one-dimensional atmospheric model and contains four variables, X_1, X_2, X_3, and X_4, and is governed by four equations:

$$dX_k/dt = -X_{k-2}\, X_{k-1} + X_{k-1}\, X_{k+1} - X_k + F$$

where k = 1, 2, 3, and 4, and F is an external forcing independent of k. X_k is considered to be the value of some unspecified scalar climatological quantity (47) such as atmospheric temperature or zonal velocity.

The variables are scaled so that the nonlinear interaction and linear damping coefficients trend toward unity, where the time unit (assumed to be 1.25 years) is scaled

by the dissipative decay time, which roughly represents the time required for the climate mode of a coupled system in the tropical Pacific to decay, as deduced from our coupled data assimilation system. For simplicity, the model is constructed with k = 4. We assume F = 8 for a control run to simulate an ENSO-like oscillator. A fourth-order Runge-Kutta scheme is used to integrate the model. The model time step was 0.01 units, or 4.56 days. We integrate forward for 200 years. This phenomenological model includes many simplifying assumptions. For example, the nonlinear term is simply expressed by quadratic terms subject to the constraint of energy conservation. Nevertheless, the model contains the components that are essential for simulating the behavior of the nonlinear climatological oscillator that is assumed to describe the tropical Pacific. It was used here to examine the behavior of a nonlinear, oscillatory system subject to repeated forcing at a well-defined periodicity.

Fig. S1 shows the temporal evolution of a model variable (such as surface zonal wind) when the tropical climate system is represented as an oscillator in unison with the ENSO timescale. Two cases are examined — one involving a constant forcing (Fig. S1A) and the other a forcing that includes sinusoidal variations with a 1-year periodicity (Fig. S1B) where F is formulated as the summation of sinusoidal functions with 1-year period and represents the "seasonal" variability:

$$F=F_1 + F_2 \sin t/T_1$$

where t is time, T_1 is 1 year, and F_1 and F_2 are 8, and 12, respectively. The ratio of F_1 to F_2 is roughly estimated from the temporal variability of zonal wind stress in the tropical Pacific.

The result in the case of constant forcing shows natural periodic behavior at the 2.5-year period inherent in the system (Figs. S1A and C), which is assumed to behave like a coupled oscillator defining the basic timescale associated with ENSO variability (2-8 years). The case of a sinusoidal forcing gives an irregular temporal evolution resulting from the interaction between the modeled seasonal and the natural periodic variations. In a basic sense, this simulates the irregular behavior of the ENSO phenomenon in the real climate system (Fig. S1B).

Wavelet analysis of the case with the sinusoidal forcing (Fig. S1D) shows clear modulation of the seasonal variability by the nonlinear interactions. While the seasonal forcing stimulates variations within a waveband centered on one year, the power of these variations changes on a decadal timescale (Fig. S1D). This quasi-decadal modulation is similar to that observed in the tropical climate (Fig. 1C) and demonstrates a modulation of the seasonal variability by nonlinear interactions in the real climate system.

S3: Ensemble forecast experiment for 2014

The numerical experiments were executed with optimized coupling parameters deduced from a coupled data assimilation experiment for the first three months. The prediction therefore starts at 1st April and 1st October 2014. The procedure used for the ensemble experiment is the same as that of the hindcast experiment in Fig 4.

Figure legends for SI:

Fig. S1: Temporal variations of a variable in the conceptual model. (A) Oscillatory temporal development with constant forcing at 2.5 years. (B) Irregular behavior with the addition of a forcing with periods of 1 year representing the seasonal variability. (C) Wavelet analysis of the variable in case (A) shows a steady limit-cycle. (D) Wavelet analysis of the variable in case (B) reveals clear modulation of both the seasonal and interannual variations that stem from self-excited variations due to the nonlinear interactions. The numerical values of the ordinate y axes for (A) and (B) denote non-dimensional units for the low-order model.

Fig. S2: **Predicted NINO 3.4 SST (red and blue curve) as compared with observed SST (black) and the accuracy of prediction.** The values are for hindcast experiments during (A) 1975–1977, (B) 1981-1983, (C) 1985-1987, (D) 1990-1992, and (E) 1996-1998 derived from the case with conventionally simulated coupling parameters (Factor = 0: blue) and the adjusted case with seasonally optimized parameters (Factor = 1: red). Units are degrees Celsius. Bars show errors estimated from standard deviation of the ensemble forecasts. Grey shaded regions denote the boreal spring periods.

Fig. S3: Extended time series of the decomposed W_{mp} spectrum at seasonal timescale over March 2012. The time series of the seasonal variability of the mean perturbation wind power in Fig. 1D is extended by using ocean state estimation from April 2006 to December 2009 (ESTOC: http://www.godac.jamstec.go.jp/estoc/e/top/) and coupled data assimilation (CDA) results from January 2010 to March 2012 with a 3-month assimilation window for recent years (48).

References for SI:

47. Lorenz, E. & Emmanuel, K. Optimal Sites for Supplementary Weather Observations. *J. Atmos. Sci.* **55**, 399-414 (1998).

48. Nishikawa, S. et al. Argo data assimilation and its effect on ocean-atmosphere coupled state estimation and forecasting in the western North Pacific. *J. Geophys. Res.* **121**, 10.1002/2014JC010095 (2015).

Fig. S1

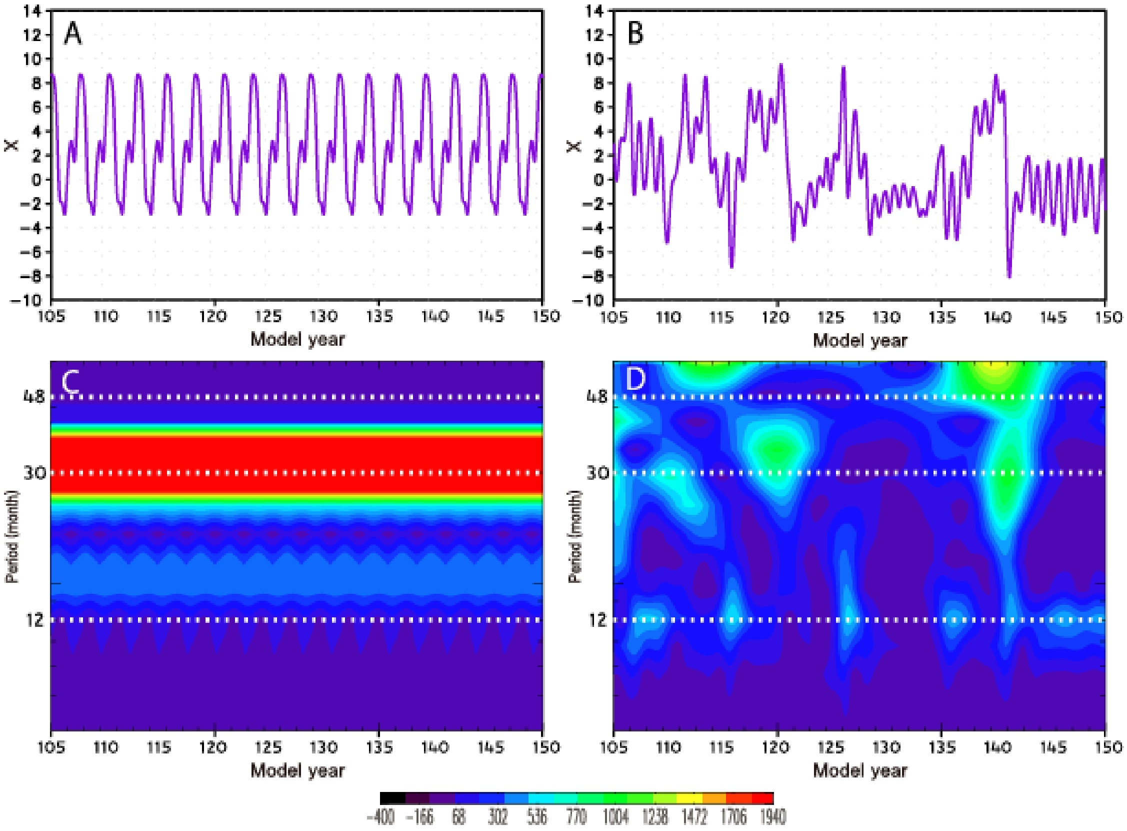
A
B
C
D
X
Model year
Period (month)
48
30
12
105 110 115 120 125 130 135 140 145 150
-400 -166 68 302 536 770 1004 1238 1472 1706 1940

Fig. S2

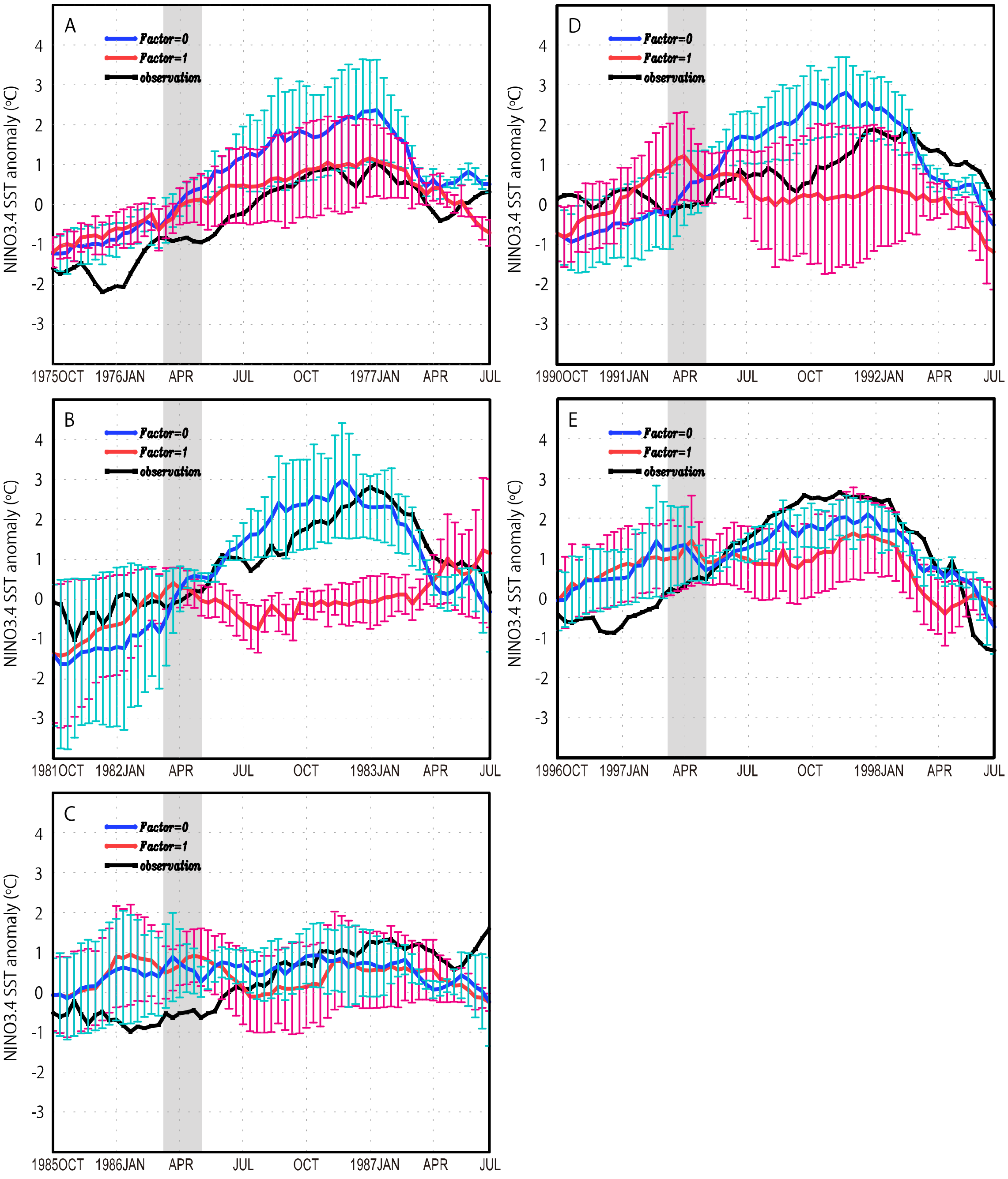
A
Factor=0
Factor=1
observation
NINO3.4 SST anomaly (ºC)
4
3
2
1
0
-1
-2
-3
1975OCT
1976JAN
APR
JUL
OCT
1977JAN
APR
JUL
D
Factor=0
Factor=1
observation
NINO3.4 SST anomaly (ºC)
1990OCT
1991JAN
APR
JUL
OCT
1992JAN
APR
JUL
B
Factor=0
Factor=1
observation
NINO3.4 SST anomaly (ºC)
1981OCT
1982JAN
APR
JUL
OCT
1983JAN
APR
JUL
E
Factor=0
Factor=1
observation
NINO3.4 SST anomaly (ºC)
1996OCT
1997JAN
APR
JUL
OCT
1998JAN
APR
JUL
C
Factor=0
Factor=1
observation
NINO3.4 SST anomaly (ºC)
1985OCT
1986JAN
APR
JUL
OCT
1987JAN
APR
JUL

Fig. S3

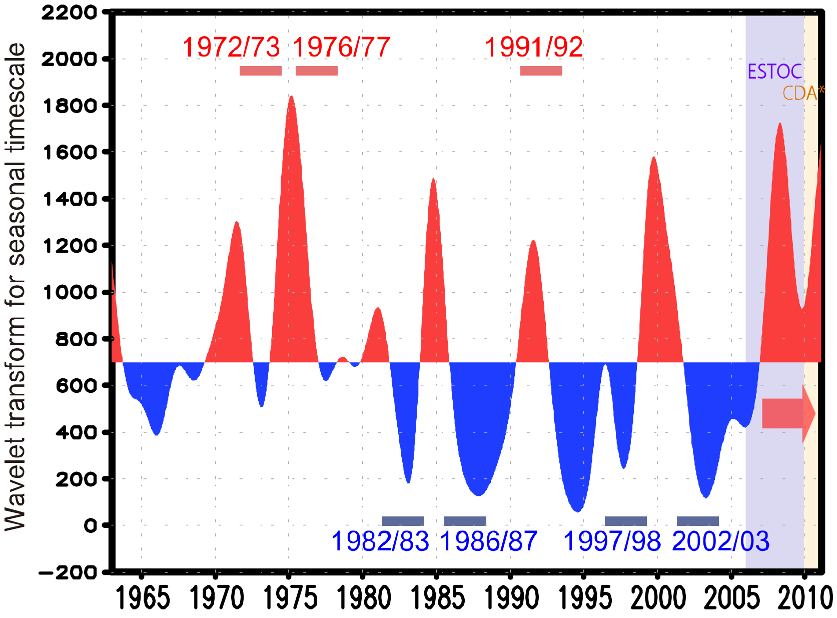

Wavelet transform for seasonal timescale
2200
2000
1800
1600
1400
1200
1000
800
600
400
200
0
−200
1965
1970
1975
1980
1985
1990
1995
2000
2005
2010
1972/73
1976/77
1991/92
1982/83
1986/87
1997/98
2002/03
ESTOC
